# The Human Sense of Smell: Are We Better Than We Think?

**DOI:** 10.1371/journal.pbio.0020146

**Published:** 2004-05-11

**Authors:** Gordon M Shepherd

## Abstract

Gordon Shepherd challenges the notion - based on genetic evidence - that olfaction is less well developed in humans as compared to other mammals

*“… a complete, comprehensive understanding of odor … may not seem a profound enough problem to dominate all the life sciences, but it contains, piece by piece, all the mysteries.” — Lewis Thomas*

One of the oldest beliefs about human perception is that we have a poor sense of smell. Not only is this a general belief among the public, but it appears to have a scientific basis. Recent genetic studies show a decline in the number of functional olfactory receptor genes through primate evolution to humans. Human evolution was characterized by the gradual ascendance of vision and reduction of smell, evidenced in the anthropological record by the progressive diminution of the snout as the eyes moved to the middle of the face to subserve depth vision (Jones et al. 1992). Concurrently, the use of an arboreal habitat and the adoption of an erect posture moved the nose away from the ground, with its rich varieties of odors.

However, some recent behavioral studies suggest that primates, including humans, have relatively good senses of smell. Resolution of this paradox may come from a larger perspective on the biology of smell. Here we begin by reassessing several overlooked factors: the structure of the nasal cavity, retronasal smell, olfactory brain areas, and language. In these arenas, humans may have advantages which outweigh their lower numbers of receptors. It appears that in the olfactory system, olfactory receptor genes do not map directly onto behavior; rather, behavior is the outcome of multiple factors. If human smell perception is better than we thought, it may have played a more important role in human evolution than is usually acknowledged.

## Gene Studies

From rodents through the primate series to humans there is a progressive reduction in the proportion of functional olfactory receptor genes (Rouquier et al. 2000; Gilad et al. 2004). Mice have approximately 1,300 olfactory receptor genes, of which some 1,100 are functional (Young et al. 2002; Zhang and Firestein 2002), whereas humans have only some 350 functional genes of approximately 1,000 (Glusman et al. 2001; Zozulya et al. 2001). The conclusion seems obvious: the low number of functional olfactory receptor genes in humans compared with rodents—and presumably most other mammals—is directly correlated with the evolutionary decline in the human sense of smell.

## Behavioral Studies

Although these conclusions seem incontrovertible, they are challenged by some recent behavioral studies. One type of study shows that much of the olfactory system can be removed with no effect on smell perception. The olfactory receptor genes map topographically onto the first relay station, a sheet of modules called glomeruli in the olfactory bulb. Up to 80% of the glomerular layer in the rat can be removed without significant effect on olfactory detection and discrimination (Bisulco and Slotnick 2003). If the remaining 20% of the glomeruli—and the olfactory receptor genes they represent—can subserve the functions of 1,100 genes, it implies that 350 genes in the human are more than enough to smell as well as a mouse.

Another type of study has tested smell perception in primates, and has shown that, despite their reduced olfactory receptor gene repertoire, primates, including humans, have surprisingly good senses of smell (Laska et al. 2000). Comparing the data on smell detection thresholds shows that humans not only perform as well or better than other primates, they also perform as well or better than other mammals. When tested for thresholds to the odors of a series of straight-chain (aliphatic) aldehydes, dogs do better on the short chain compounds, but humans perform as well or slightly better than dogs on the longer chain compounds, and humans perform significantly better than rats (Laska et al. 2000). Similar results have been obtained with other types of odors.

A third type of study demonstrating human olfactory abilities shows that in tests of odor detection, humans outperform the most sensitive measuring instruments such as the gas chromatograph.

These results indicate that humans are not poor smellers (a condition technically called microsmats), but rather are relatively good, perhaps even excellent, smellers (macrosmats) (Laska et al. 2000). This may come as a surprise to many people, though not to those who make their living by their noses, such as oenologists, perfumers, and food scientists. Anyone who has taken part in a wine tasting, or observed professional testing of food flavors or perfumes, knows that the human sense of smell has extraordinary capacities for discrimination.

## The Mystery

Here, then, is the mystery: how can one reconcile a relatively high sensitivity to smell with a relatively low number of olfactory receptors in the nose? To answer this question, I think we need to look beyond the olfactory receptor genes and consider olfaction in its full behavioral context. This requires considering several overlooked aspects of the olfactory system: the nasal cavity, the oropharyngeal cavity, the olfactory brain, and the role of language. In this article I focus on behaviors related to conscious perception of ordinary smells. Pheromones, and the rich world of unconscious effects of odors and pheromones, are beyond the present scope (cf. Jacob et al. 2004), though they undoubtedly will add to the general conclusions.

## The Filtering Apparatus of the Nasal Cavity

A marked difference between the noses of primates and other mammals is that in nearly all nonprimate mammals, the nasal cavities contain at the front a much-convoluted filtering apparatus (formed by the ethmo- and maxillo-turbinals) covered with respiratory membrane. This filtering apparatus is a biological air conditioner (Negus 1958) with three key functions: cleaning, warming, and humidifying the inspired air. An important function of the filtering apparatus is presumably to protect the nasal cavity from infections. In many mammals, air drawn into the nose is often highly contaminated with bacteria from fecal material, decaying animal and plant material, and noxious fumes from the environment, all of which attack the olfactory epithelium. Rodents are susceptible to chronic rhinitis, which causes substantial loss of functioning olfactory receptor cells (Hinds et al. 1984).

This filtering, however, might have negative consequences for odor detection. Warming and humidification presumably enhance the odor-stimulating capacity of the inhaled air, but cleaning would remove odor molecules by absorbing them into the lining of the epithelium, an effect which could be large depending on the size of the filtering apparatus. If so, mammals with large snouts might have a large inventory of olfactory receptors at least in part to offset the loss of odor molecules absorbed by the filtering apparatus.

How do these considerations relate to humans? The evolution of humans involved lifting the nose away from the noxious ground environment as they adopted a bipedal posture (Aiello and Dean 1990). This would have reduced the need for the filtering apparatus and with it the losses of absorbed odor molecules. The large numbers of olfactory receptors and receptor cells would have come under reduced adaptive pressure and could accordingly be reduced in proportion.

By this hypothesis, during human evolution the snout could be reduced in dimensions and complexity without compromising the ultimate amounts of odorized air reaching the olfactory epithelium. The reduced snout allowed the eyes to come forward and lie closer together to promote more effective stereoscopic vision. Thus, vision could become more dominant in humans without sacrificing unduly the sense of smell. Tests of this hypothesis are needed, including calculations of air flows and odor losses through the filtering apparatus in mammals with extensive filtering apparatuses compared with the simpler nasal cavities of primates.

## Humans Receive Richer Retronasal Smells

Being carried in with inhaled air (the orthonasal route) is not the only way for odor molecules to reach the olfactory receptor cells. Odor molecules also reach the olfactory receptor cells via the retronasal route, from the back of the oral cavity through the nasopharynx into the back of the nasal cavity. Although the orthonasal route is the one usually used to test for smell perception, the retronasal route is the main source of the smells we perceive from foods and liquids within our mouths. These are the smells that primarily determine the hedonic (i.e., pleasurable or aversive) qualities of foods, and that, combined with taste and somatosensation, form the complex sensation of flavor. It is likely, for several reasons, that this is an important route for smell in humans.

First, with the adoption of bipedalism, humans became increasingly wide ranging, with concomitant diversification of diet and retronasal smells. Second, the advent of fire, perhaps as early as 2 million years ago (Wrangham and Conklin-Brittain 2003), made the human diet more odorous and tasty. From this time also one can begin to speak of human cuisines of prepared foods, with all their diversity of smells. Wrangham and Conklin-Brittain (2003) support the view that prepared cuisines based on cooked foods are one of the defining characteristics of humans. Third, added to the cooked cuisines were fermented foods and liquids, with their own strong flavors. These developments occurred among the early hunter-gatherer human cultures and continued through the last ice age. With the transition to agricultural and urban cultures 10,000 years ago, human cuisines changed by the advent of animal domestication, plant cultivation, use of spices, and of complex procedures, such as those for producing cheeses and wines, all of which produced foodstuffs that especially stimulate the smell receptors in the nose through the retronasal route and contribute to complex flavors.

**Figure 1:**
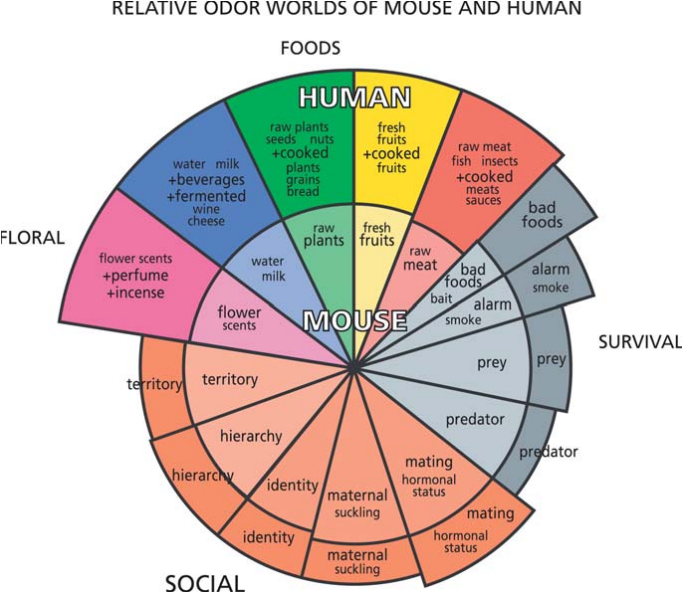
Hypothetical “Odor Wheel” Representing and Comparing the Odor Worlds of Mouse and Human The inner part represents the different categories of odors for the mouse; the relative importance of each category for mouse smell-dependent behavior is indicated by the area of each wedge. The outer part represents the same categories for the human; the importance of each category for human smell-dependent behavior compared with the mouse is indicated by the area of each wedge. Note the greater importance of food odors for the human, reflecting the factors discussed in the text. Note also the retention of some sensitivity in humans to social odors and other odors prominent in rodents, though in many cases to still undetermined degrees. Based on numerous sources and the hypotheses discussed in the text.

These considerations suggest the hypothesis that the retronasal route for smells has delivered a richer repertoire of smells in humans than in nonhuman primates and other mammals (see Figure 1). Research on retronasal olfaction is being actively pursued (reviewed in Deibler and Delwiche 2004). Studies are needed of the evolutionary pressures on this route in addition to the pressures on the evolution of the snout.

## Humans Smell with Bigger and Better Brains

Comparisons of the decreasing size of the olfactory system relative to expansion of the visual, auditory, and somatosensory systems usually focus on the olfactory bulb and lateral olfactory tract, which are relatively small. However, what matters more are the central olfactory brain regions that process the olfactory input as the basis for smell perception.

These regions are more extensive in humans than is usually realized. The dedicated olfactory regions include the olfactory cortex, the olfactory tubercle, the entorhinal cortex, parts of the amygdala, parts of the hypothalamus, the mediodorsal thalamus, the medial and lateral orbitofrontal cortex, and parts of the insula (Neville and Haberly 2004). These regions are involved in immediate processing of odor input and probably subserve the specific tasks of smell detection and simple smell discrimination. For more complex tasks, memory becomes important in comparing smells, thus involving the temporal and frontal lobes (e.g., Buchanan et al. 2003) and the specifically human higher association areas. It may be hypothesized that these regions enable humans to bring far more cognitive power to bear on odor discrimination than is possible in the rodent and other mammals.

The reduced repertoire of olfactory receptor genes in the human is thus offset by the expanded repertoire of higher brain mechanisms. Rather than being restricted to a tiny part of the brain, olfactory processing of complex smells, such as those produced by human cuisines, draws on the enlarged processing capacity of the human brain.

## Language Is Necessary for Human Smell

In the enlarged processing capacity for perceiving and discriminating odors, language plays a critical role. This seems paradoxical, for we have great difficulty describing a smell in words. Insight into this difficulty comes from the finding that different smells are represented in the olfactory bulb by different patterns of olfactory glomerular activity. These patterns function as virtual “odor images” (Xu et al. 2003). It has been hypothesized that these odor images provide the basis for discrimination between odors, analogous to the way that retinal images are the basis for discrimination of visual pattern stimuli. The complex patterns constituting odor images may be considered as analogous to the complex patterns constituting visual images of faces. And just as we are very good at recognizing a human face, yet have difficulty describing it in words, we have a hard time describing and verbally comparing odor images.

Because of this difficulty, describing a smell or a taste in words is very demanding. A professional wine tasting, for example, requires many steps: analysing both orthonasal and retronasal perception, comparing the two in memory with each other and with all other wines to be compared, identifying the constituent properties separate from the hedonic qualities, and finding the words to describe the process as it unfolds, leading to the final formulation to characterize the quality of the wine and identify it as distinct from all others. It may be characterized as hard cognitive work that only a human, among all the animals with olfactory organs, can do. It may be argued that this is what humans are adapted to do (Wrangham and Conklin-Brittain 2003).

This cognitive work is largely independent of the numbers of peripheral receptor cells and their genes. A good analogy is with language. There are some 17,000–20,000 auditory nerve fibers in the rat and cat and some 25,000–30,000 in the human (cf. Hall and Masengill 1997). This modest increase in the input from the peripheral auditory receptors provides little basis for the development of human speech and language, which had much more to do with the increase in the central brain mechanisms that elaborate the input. It may be hypothesized that a similar conclusion applies to human olfaction.

## Implications for Systems Biology

A general result from these considerations is that there appears not to be a one-to-one relation between the number of olfactory receptor genes and the detection and discrimination of odors. This implies that we are dealing with a fundamental problem in relating genes to systems behavior: a given set of genes may not map directly onto a given behavior. In this respect the mystery being addressed here is a caution for the new era of “systems biology” and against any belief that behavior can be related directly to genomes, proteomes, or any other type of “-ome.” We are reminded instead that the functional ecology of the body is dependent on many factors.

## Conclusions

Much about the sense of smell seems enigmatic and conflicting. This is partly because of the inherent difficulties in presenting smell stimuli, and partly because there is not yet a recognition of all the relevant mechanisms that are involved.

It may be hoped that the hypotheses and mechanisms discussed here can help to address and resolve the mystery of the apparent noncorrelation of olfactory receptor gene numbers with smell acuity, and in doing so stimulate a major reassessment of human smell perception. Such an effort cuts across many academic disciplines. Molecular biologists need to continue their efforts to characterize the olfactory genomes of humans and nonhuman mammals more closely, to compare how different organisms sample odor space. Physiologists need to devise high-throughput systems to test these odor spaces. Behavioral neuroscientists need to develop increasingly accurate tests of olfactory function that enable comparisons across different species. Psychologists need to explore even more vigorously the subtle ways that smells can influence human behavior. Anthropologists and paleontologists need to study the olfactory parts of the cranium and face from this new perspective, to reassess the role that both orthonasal and retronasal smell may have played in primate and human evolution.

The factors reviewed here suggest that the sense of smell is more important in humans than is generally realized, which in turn suggests that it may have played a bigger role in the evolution of human diet, habitat, and social behavior than has been appreciated. All of these considerations should stimulate a greater interest in this neglected sense.

## References

[pbio-0020146-Aiello1] Aiello L, Dean C (1990). An introduction to human evolutionary anatomy.

[pbio-0020146-Bisulco1] Bisulco S, Slotnick B (2003). Olfactory discrimination of short chain fatty acids in rats with large bilateral lesions of the olfactory bulbs. Chem Senses.

[pbio-0020146-Buchanan1] Buchanan TW, Tranel D, Adolphs R (2003). A specific role for the human amygdala in olfactory memory. Learn Mem.

[pbio-0020146-Deibler1] Deibler KD, Delwiche J (2004). Handbook of flavor characterization: Sensory analysis, chemistry, and physiology.

[pbio-0020146-Gilad1] Gilad Y, Wiebe V, Przeworski M, Lancet D, Pääbo S (2004). Loss of olfactory receptor genes coincides with the acquisition of full trichromatic vision in primates. PLoS Biol.

[pbio-0020146-Glusman1] Glusman G, Yanai I, Rubin I, Lancet D (2001). The complete human olfactory subgenome. Genome Res.

[pbio-0020146-Hall1] Hall RD, Massengill JL (1997). The number of primary auditory afferents in the rat. Hear Res.

[pbio-0020146-Hinds1] Hinds JW, Hinds PL, McNelly NA (1984). An autoradiographic study of the mouse olfactory epithelium: Evidence for long-lived receptors. Anat Rec.

[pbio-0020146-Jacob1] Jacob S, Spencer NA, Bullivant SB, Sellergren SA, Mennella JA (2004). Effects of breastfeeding chemosignals on the human menstrual cycle. Hum Reprod.

[pbio-0020146-Jones1] Jones S, Martin R, Pilbeam D (1992). The Cambridge encyclopedia of human evolution.

[pbio-0020146-Laska1] Laska M, Seibt A, Weber A (2000). “Microsmatic” primates revisited: Olfactory sensitivity in the squirrel monkey. Chem Senses.

[pbio-0020146-Negus1] Negus V (1958). The comparative anatomy and physiology of the nose and paranasal sinuses.

[pbio-0020146-Neville1] Neville KR, Haberly LB, Shepherd GM (2004). Olfactory cortex. The synaptic organization of the brain, 5th ed.

[pbio-0020146-Rouquier1] Rouquier S, Blancher A, Giorgi D (2000). The olfactory receptor gene repertoire in primates and mouse: Evidence for reduction of the functional fraction in primates. Proc Natl Acad Sci U S A.

[pbio-0020146-Wrangham1] Wrangham R, Conklin-Brittain N (2003). Cooking as a biological trait. Comp Biochem Physiol A Mol Integr Physiol.

[pbio-0020146-Xu1] Xu F, Liu N, Kida I, Rothman DL, Hyder F (2003). Odor maps of aldehydes and esters revealed by functional MRI in the glomerular layer of the mouse olfactory bulb. Proc Natl Acad Sci USA.

[pbio-0020146-Young1] Young JM, Friedman C, Williams EM, Ross JA, Tonnes-Priddy L (2002). Different evolutionary processes shaped the mouse and human olfactory receptor gene families. Hum Mol Genet.

[pbio-0020146-Zhang1] Zhang X, Firestein S (2002). The olfactory receptor gene superfamily of the mouse. Nat Neurosci.

[pbio-0020146-Zozulya1] Zozulya S, Echeverri F, Nguyen T (2001). The human olfactory receptor repertoire. Genome Biol.

